# Special Issue on Advanced Sensing and Control Technologies in Power Electronics

**DOI:** 10.3390/s25103230

**Published:** 2025-05-21

**Authors:** Krzysztof Bernacki, Mateja Novak

**Affiliations:** 1 Department of Electronics, Electrical Engineering and Microelectronics, Silesian University of Technology, Akademicka 16, 44-100 Gliwice, Poland; 2 Department of Energy Technology, Aalborg University, 9220 Aalborg, Denmark; nov@energy.aau.dk

## 1. Introduction

Power electronics performs a key role in today’s energy systems, and its development is increasingly combined with advanced sensor-critical techniques and novel control algorithms that significantly enhance system adaptability, reliability, and robustness against disturbances and cyber threats. In the era of the increasing use of renewable energy sources, storage systems, and distributed power systems, there is an emerging need for the accurate measurement of electrical parameters and sophisticated control to ensure stability, efficiency, and robustness against disturbances. This Special Issue of the *Sensors* Journal, titled Advanced Sensing and Control Technologies in Power Electronics, brings together sixteen articles presenting the latest developments in these areas. The publications range from new sensing solutions such as optimized sensors for leakage current measurements, electromagnetic detection systems, and advanced vision systems, to innovative control methods for power electronics systems such as predictive and adaptive controllers, sensor-less motor control, and fault-tolerant and cyber-resilient systems, and algorithms for controlling motors and converters to strategies for making systems more resilient to disruptions and cyber-attacks. The published works are reviewed below, indicating their main ideas, the methods used, and the key conclusions, in the context of the common theme of the Special Issue.

## 2. Review of the Published Works

### 2.1. Measurement and Sensor Technologies in Power Electronics

Several of the Issue’s papers focus on improving the measurement apparatuses used in power electronics systems. Tan et al. [1] proposed a novel secondary fast-discharge circuit for a pulsed electromagnetic transmitter (TEM). Adding an additional RLC circuit (with a capacitor, resistors, and MOSFET transistors) to the classical H-bridge structure made it possible to reduce the current-off time in the transmitter’s inductive coil to about 64 µs (regardless of the current amplitude), and simultaneously significantly reduced the overvoltage across the MOSFET transistors, thus protecting them from potential damage [1]. This solution significantly improves the characteristics of the transient electromagnetic method used, for example, in geophysics, enabling the better reception of secondary signals from the ground. Complementing this topic is an article by Liao et al. [2], which presents a complete electromagnetic detection system with a moving magnetic dipole for near-surface surveys. The authors designed a non-invasive TEM measurement system with a separated transmitter and receiver: a heavy transmitter generating strong magnetic pulses was separated from a lightweight, portable receiver module, effectively minimizing interference and enhancing measurement accuracy [2]. A bipolar resonant pulse power supply was used to achieve the transmitter’s high magnetic moment with reduced interference levels. In field tests, the system was able to quickly scan an area up to 200 m from the transmitter, effectively detecting shallow objects [2]. Another aspect of advanced sensors is discussed in a paper by Hu et al. [3], concerning a multistage TMR (magneto-resistive tunnel effect) sensor for measuring very small AC/DC leakage currents in electric power equipment. The authors designed an array of multi-ring magnetic cores to increase the measurement sensitivity and resistance to external interference fields [3]. The optimization of the shape and size of the multipole core was carried out using a genetic algorithm, directly contributing to the sensor’s high sensitivity and improved immunity to external interference fields, obtaining a sensor structure capable of measuring currents of tens of µA with a wide bandwidth (0–80 kHz) and nonlinearity < 1% [3]. The experimental results confirmed the high precision and stability of this sensor even in a highly interfering electromagnetic environment, which is important for network health monitoring and energy security. In turn, Li et al. [4] presented a solution that goes beyond traditional electrical measurements—they developed an active system for tracking multiple moving objects using a high-speed imaging camera and vision algorithms. The system, operating at 500 frames/s, combines a hardware galvanometric lens control module (galvano-mirror module allowing dynamic and precise lens control) with a hybrid object detection algorithm based on a convolutional neural network (CNN) [4]. A real-world demonstration showed the ability to simultaneously track up to three objects moving at speeds of up to 30 m/s within an 8 m range, while maintaining high image resolution through dynamic zoom [4]. This solution illustrates the growing synergy between sensory systems (here, high-speed vision systems) and control systems, where acquired vision data can feed control algorithms in applications such as robotics or autonomous device control.

### 2.2. New Control Methods for Drives and Converters

Most of the Issue’s articles focus on improved control algorithms for power electronic systems, increasing their efficiency, operating range, and reliability. Guo et al. [5] proposed a four-stage method for starting a sensor-less synchronous reluctance motor (SynRM) based on current and frequency control (the so-called I-f method) with additional inductance identification and high-frequency voltage injection. The goal was to provide a smooth transition through the critical low-speed range, where classical back-EMF observation algorithms fail due to a negligibly low flux [5]. The simple inductance saturation identification used and the adaptive forcing of the HF component of the current allowed for the control of the current amplitude and rotor position during start-up, eliminating the need for mechanical position sensors and providing a significant technological advancement, ensuring a stable SynRM start-up and the subsequent transition to the sensor-less mode. Experiments on a 5.5 kW motor confirmed the effectiveness of the method—the reliable estimation of rotor position at low speeds was obtained and the dynamics of start-up were improved [5]. Praženica et al. [6], however, focused on the practical implementation of converter control for a multi-phase drive. The subject of their work is a 3×5-phase direct array converter (AC/AC), converting 3-phase voltage into 5-phase voltage, capable of powering a 5-phase drive. The authors implemented an algorithm for the so-called indirect control of such a converter—treating it conceptually as a separate rectifier and inverter by using an inexpensive DSP chip working with a programmable FPGA array [6]. Due to the limited number of PWM outputs in the DSP itself and the need for the very fast and safe co-mutation of 15 transistor devices, the key timing tasks (the generation of control signals and transient protection) were moved to the FPGA logic, while the higher-level control algorithm was implemented in the DSP, demonstrating the practical benefits of combining cost-effective FPGA and DSP chips for advanced converter control [6]. This separation made it possible to reduce the cost of the control system, while providing precise control of the waveforms. The built prototype confirmed that the adopted control strategy allows for wide output frequency control and power factor control on the input side, which demonstrates the usefulness of 3 × 5 converters in multi-phase drives and the validity of using low-cost DSP+FPGA platforms in advanced power electronics control [6]. Publication [7] presents a new adaptive control strategy for a bidirectional DC-DC converter, combining the sliding mode control technique with an extremum-seeking control algorithm. The purpose of the proposed method was to ensure stable operation and high-quality control under conditions of varying system parameters and unknown disturbances. The extremum-seeking algorithm was used for the adaptive tuning of the sliding surface parameters, which allows for the dynamic adjustment of the controller characteristics without knowing the exact model of the converter. The authors conducted detailed stability analyses of the system and confirmed the effectiveness of the approach in both simulation and experimental studies. The results show that the proposed control provides fast tracking of the operating point, high robustness to model uncertainty, and good quality of the dynamic response [7].

### 2.3. Single-Phase Voltage Converters

Another group of papers deals with the single-phase voltage converters (inverters) used in UPS or photovoltaic systems, among others, where both high-quality output waveforms and robustness to load changes are important. Andrea et al. [8] presented a methodology for designing a model-based controller (MPC) for a single-phase LC-filtered inverter, taking into account the uncertainty of the system parameters. The problem of ensuring the stable regulation of sinusoidal voltage at the output of the converter under varying loads was solved by formulating stability conditions in the form of linear matrix inequality (LMI) constraints, which were then solved via convex optimization methods [8]. The designed predictive controller with an additional integral member (crucial for robust stabilization under uncertain system parameters) effectively tracked the sinusoidal signal and suppressed disturbances (supply voltage fluctuations and load changes), as demonstrated by MATLAB/Simulink simulations and hardware-in-the-loop tests on an FPGA chip [8]. In another paper, Li et al. [9] proposed a predictive control strategy without current sensors for a single-phase UPS-type inverter. The key element here is a time-varying load current observer that allows for the accurate estimation of both the fundamental component and the higher harmonics of the periodic current [9]. The load current thus estimated (including the accurate estimation of higher harmonic components) is used in the Finite Control Set MPC algorithm to control the inverter’s devices, eliminating the need for a physical output current sensor. A comparison with classical sensor-less methods (low-pass filter and Kalman observer) showed that the proposed observer provides lower current estimation error and lower output voltage distortion (THD) for both linear and nonlinear loads [9]. Complementing the above work is an article by Bernacki and Rymarski [10], which presents a cross-sectional, contemporary design process for single-phase voltage inverter control systems. The authors reviewed the current design methods and criteria—from mathematical models and classical PI/PR coupled control to advanced techniques based on state observers or predictive control—to formulate consistent design guidelines for engineers [10]. The importance of already considering physical constraints (e.g., converter saturation and discretization) at the pro-design stage is emphasized, and simulation tools to help optimize the regulator settings are discussed. This holistic approach provides valuable guidance, complementary to the work of [8,9], and promotes the transfer of the latest scientific advances for practical implementation in industrial power electronics systems.

### 2.4. Topologies and Control Strategies of Converters

A separate category is made up of publications proposing innovative topologies and control strategies for converters, expanding their functionality and range of stable operation. Tirupathi et al. [11] focused on a three-phase hybrid cascaded H-bridge (HCHB) inverter with multi-voltage sources, used in medium-voltage systems, among others. The problem with such systems is the drift of capacitor voltages in the intermediate circuits at certain operating points (e.g., low loads or asymmetrical power factors), which limits the permissible modulation range. The paper [11] proposes a generalized control scheme that extends the operating range of the HCHB inverter without requiring hardware modifications. It involves introducing two independent offset components into the modulation reference waveforms to simultaneously control the voltages on DC-link capacitors and flying capacitors [11]. In addition, by dynamically adjusting the common zero component of the reference signal to the nearest voltage level, a limited number of redundant states are compensated for at low modulation indices. Simulations and experimental tests (on a prototype) have confirmed that this control strategy effectively maintains capacitor voltages within the assumed range for various load and modulation conditions, significantly improving the system’s ability to operate at a wider range of output voltages [11]. Another proposal was presented by Obi et al. [12], focusing on a single-phase, multifunctional AC/DC converter with galvanic isolation. The topology presented here is based on a single center-tapped transformer connecting the full-bridge converter to the grid. This transformer plays a dual role: for example, it provides the galvanic isolation required by the standards when connecting the energy storage to the grid, and at the same time, its dissipation inductance serves as a filtering and limiting element in the current circuit [12]. By using wide DC-bus control, the circuit can operate as a single-phase rectifier or inverter over a very wide range of input/output voltages, performing functions in a single conversion stage that would traditionally require two stages (e.g., a separate DC/DC boost and DC/AC inverter). The authors indicate that by cascading several such modules, a multilevel structure can be created to improve the output voltage quality, highlighting the multifunctional capabilities (rectification, inversion, and galvanic isolation) achievable with a single hardware configuration [12]. Simulation tests have been carried out for a 1.8 kVA prototype (220 V, 45–100 Hz) and small-scale experiments (60–100 V), which have confirmed the system’s post-operational bidirectional operation (both AC load supply from the battery and battery charging from the grid) and the effectiveness of maintaining a stable DC-link voltage over a wide range of conditions [12].

### 2.5. Fault-Tolerant Control to Improve Reliability

An important trend in power electronics is to ensure the stable operation of systems under conditions of disturbances, failures, or cyber-attacks. This Special Issue features papers proposing solutions to make systems more resilient to such undesirable factors. Ma et al. [13] addressed fault detection in distributed DC microgrids under DoS (denial of service) cyber-attacks. They proposed a resilient fault detection algorithm based on fuzzy logic and event-based triggering in the finite frequency domain [13]. The system model was based on the T-S (Takagi–Sugeno) fuzzy representation, which made it possible to take into account the nonlinearity of microgrids with loads with constant power characteristics. A key innovation is the introduction of an integrated event-triggering mechanism (ETM), which significantly enhances system resilience against data loss due to DoS attacks, because unlike the classical approach based solely on current samples, it also uses historical information about post-measurement signals [13]. This approach reduces the frequency of unnecessary transmissions (the so-called data releasing rate) caused by random fluctuations, while maintaining sensitivity to significant changes indicating damage. For the assumption that damage manifests in a limited frequency band, a failure detection filter (FDF) with a generalized H∞ criterion, resistant to missing data caused by a DoS attack, was developed. The stability conditions of the system with the detection filter were formulated as inequalities, and simulations showed that the designed fuzzy observer effectively and quickly detected damage in the set band even with periodic losses of communication, surpassing the sensitivity of classical detection methods [13]. The issue of resilience to cyber-attacks in HVDC converter systems is addressed by Ramadhan et al. [14]. They focus on the so-called multi-infeed HVDC (MIDC) system, where multiple DC connections feed into a common AC network, carrying out the inter-area exchange of power and system services (e.g., frequency reserve). The authors analyzed a frequency reserve replacement scenario (E-FCR) between two areas of the system connected by several HVDC lines—such regulation is based on remote frequency measurements and the control of HVDC reserve resources [14]. A single-point E-FCR measurement architecture has been shown to be susceptible to false data/information (FDI) attacks, such as the delay or substitution of erroneous frequency readings. In [14], a coordinated defense scheme was proposed that treats the simultaneous occurrence of a network failure and a cybernetic attack as a complex event. Different variants of the attacker’s access to system data and the corresponding possible effects on frequency stability are presented. An attack detection strategy based on the normalized correlation of frequency signals from individual HVDC links is then implemented to detect the measurement anomaly [14]. Once an attack is detected, a mitigation algorithm is activated, which, by adapting the gradients (rates of change) of the HVDC component control, demands the distribution of reserves between links. Simulations of the full Korean system model in PSS/E have shown that the proposed method effectively identifies a cyber-attack masking measurement data and will activate preventive control, limiting the frequency deviation during the disturbance [14].

### 2.6. Increasing the Robustness of Control Systems to Disturbances

Other work has focused on increasing the robustness of control systems to physical disturbances and ensuring the continuity of operation during grid failures. Baskys [15] proposed an original solution to the problem of the sensitivity of classical PID controllers to electromagnetic interference (EMI) in feedback systems. The author presented a combined control system consisting of three controllers (PID, PI, and I), of which only one is active at any given time—the selection is made automatically depending on the current value of the control deviation [15]. Intuitively, for small deviations (when the measurement noise is comparable to the signal), the simpler I or PI regulator that is less sensitive to noise is preferred, while for larger deviations, the full PID is switched, providing a fast dynamic response. Such adaptive switching preserves the advantages of PID (speed and accuracy) while suppressing the effects of EMI noise due to adaptive switching between controllers depending on the magnitude of the error signal at the controller input [15]. Simulations and experiments with a delayed inertial object have shown that this approach provides smaller control fluctuations and output responses in the presence of disturbances compared to a classical PID with a low-pass filter in the D path, without degrading the quality of control in dynamic states [15]. Saeed et al. [16], on the other hand, addressed the problem of the fault ride-through (FRT) capability of DFI generators in wind power plants. DFI (doubly fed induction) generators are particularly vulnerable to the effects of grid voltage collapses—a sudden drop in the stator voltage induces strong over voltages and currents in the rotor circuit, threatening the power converters. The paper [16] proposes a hybrid protection solution: the combination of a Modified Switch-Type Fault Current Limiter (MSTFCL) in the rotor circuit and an active DC chopper in the converter’s intermediate circuit [16]. The MSTFCL uses a high-speed solid-state switch and passive components (inductance, resistor, and snubber circuit) to immediately turn on the rotor circuit when a collapse is detected—this reduces the flow of the surge current and limits the formation of an anti-electromotive force in the rotor. At the same time, the DC chopper protects the DC bus capacitor from an excessive voltage rise by dissipating the excess energy. Comparative simulations have shown that this combination effectively limits rotor currents and stabilizes the DC-link voltage during symmetrical and asymmetrical collapses, emphasizing the superiority of this hybrid hardware/software solution in ensuring fault ride-through capability, ensuring that the FRT criteria are met without disconnecting the turbine from the grid [16]. Moreover, a comparison with control-only approaches (e.g., advanced torque-tracking regulators) showed the advantages of the hybrid solution: a reduction in electromagnetic torque oscillations during failures and faster system recovery after collapse [16]. These results demonstrate that the integration of hardware components with intelligent control (as in [16]) significantly increases the resilience of power electronic systems to severe grid disturbances.

## 3. Summary and Conclusions

The work presented in this Special Issue reflects the drive to increase the complexity, autonomy, and robustness of power electronic systems through the integration of advanced measurement and control methods, especially emphasizing the importance of resilient and adaptive solutions capable of autonomous responses to changing operating conditions and external threats. From the perspective of sensor techniques, there is a noticeable trend toward the design of highly specialized sensors (such as multi-stage TMR sensors [3]) and entire measurement systems (such as mobile TEM systems [2]) tailored to specific diagnostic and control tasks in the power industry. In parallel, regarding control algorithms, approaches emphasizing adaptability and resilience dominate, such as predictive controllers capable of operating under uncertain parameters [8,9], hybrid strategies combining different control schemes [15], or observer and filter structures that allow control to be maintained even under communication disturbances [13,14]. Many of the proposed solutions are universal in nature and can find application in various branches of power electronics; for example, the method of sensor-less motor starting [5] is part of the broader trend of eliminating mechanical sensors in electric drives. The complex solution consisting of FPGAs/DSPs [6] reflects the general trend toward the use of low-cost, programmable platforms in the control of complex converters. It is worth noting the thematic synergy of the papers included in the Issue. Several papers combine measurement and control issues—for example, refs. [9,13] demonstrate how a properly designed observer (soft sensor) improves the performance of a predictive control or fault detection algorithm. Other works suggest the complementarity of hardware and software approaches: hybrid solutions (such as the limiter with control in [16]) show the most promise in providing system robustness in extreme conditions. Moreover, almost all studies emphasize experimental or simulation verification under realistic conditions—from prototype testing in the lab [1,11,12] to advanced system simulations [14,16]. This demonstrates the maturity of the proposed methods and their readiness for implementation. The achievements collected in this Special Issue are a valuable contribution to both the theory and practice of power electronics, highlighting that the synergy between advanced sensing techniques and innovative control strategies represents a foundational pillar for the next generation of robust, efficient, and intelligent power electronics systems. A common conclusion from this work is that the integration of advanced sensors (both physical and virtual) with control algorithms makes it possible to build power electronic systems with a higher level of efficiency—able to independently adapt to changing conditions, diagnose abnormal states, and maintain safe operation despite disturbances. The further development of these trends can be expected in the future. Potential research directions include but are not limited to the following: the use of artificial intelligence and machine learning to synthesize even more autonomous control systems; the integration of sensory functions into the power elements themselves (so-called smart power devices) for the direct monitoring of their conditions; and the development of standardized control architectures resistant to cyber threats for critical power infrastructures. Moreover, the consistent emphasis across the reviewed studies on rigorous experimental validation and practical feasibility confirms the immediate applicability of these innovations in industrial and commercial contexts, which further accelerates their adoption in real-world power systems. The work presented here lays a solid foundation for these activities, demonstrating that combining novel sensory solutions with adaptive and resilient control is the key to the next generation of efficient and reliable power electronics systems.
